# An integrated proteomics and transcriptomics analysis highlights concordance between protein turnover and carbohydrate transport and metabolism as key functional categories during the growth of *Trichophyton rubrum*

**DOI:** 10.1128/spectrum.03483-25

**Published:** 2026-08-04

**Authors:** Himani Singh, Chitra Latka, Rahul Chakraborty, Leepakshi Dhingra, Sherry Bhalla, Bhupesh Taneja

**Affiliations:** 1 CSIR-Institute of Genomics and Integrative Biology 28570 , New Delhi, India; 2 Academy of Scientific and Innovative Research (AcSIR) 550336, Ghaziabad, India; University of Delhi, Delhi, India

**Keywords:** dermatophytes, multi-omics data, mass spectrometry, transcriptomics, antifungal resistance

## Abstract

Dermatophytes are a class of keratinophilic skin fungi that invade host skin, hair, and nails to acquire nutrients. An integrated multi-omics approach utilizing liquid chromatography-tandem mass spectrometry and RNA-seq after growth in a protein-rich soy medium was employed to capture the major subset of secreted protein families of *Trichophyton rubrum*. The secretome consisted mainly of proteases and cell wall-degrading enzymes, with subtilisins (Sub6 and Sub7), metallopeptidase (LAP2), and chitinase having the most abundant peptides. Transcriptional profiling indicated fungal adaptation in protein-rich media to process the protein nutrients through modulation of metabolism and general cellular function pathways. Correlation analysis between proteomics and transcriptomics data using functional KOG categories shows high concordance of KOG categories O (posttranslational modification, protein turnover, and chaperones), P (inorganic ion transport and metabolism), and G (carbohydrate transport and metabolism), as per cosine similarity analysis.

**IMPORTANCE:**

Dermatophytes are keratinophilic skin fungal pathogens that invade host skin, hair, and nails to acquire nutrients. There is an epidemic-like increase in infections, as well as an increase in antimicrobial resistance among dermatophytes, as witnessed over the last decade. There is hence a need to understand the key pathways and virulence factors required during growth and infection. We present an integrated multi-omics analysis (proteomics and transcriptomics data) using a vector-based similarity approach to show high concordance of KOG functional categories belonging to posttranslational modification, protein turnover, carbohydrate transport, and metabolism.

## OBSERVATION

Dermatophytes encompass keratinophilic skin pathogenic fungi responsible for more than three-fourths of superficial fungal infections worldwide and are associated with considerable pain, morbidity, and socioeconomic trauma in the affected individuals (1, 2). A recent epidemic-like increase in infections and antimicrobial resistance among dermatophytes, as witnessed over the last decade, further compounds the problem (3, 4). Among dermatophytes, the anthropophilic fungus *Trichophyton rubrum* is the most common pathogen, responsible for 60%–70% of all infections (3, 5). During infection, dermatophytes encounter low skin pH (pH 4.5–5.5) and limited nutrition in the keratinized tissue (6). Dermatophytes are hence known to secrete a wide variety of enzymes that aid growth, adhesion, invasion, and immune evasion during infection (7–10). Among the major secreted proteins, subtilisins (Sub6 and Sub7), leucine aminopeptidase (LAP2), DppV, glucan 1,3-beta-glucosidase, and beta-glucosidase were identified in patients infected with *T. rubrum* (11), while Sub6, Sub7, Sub8, Sub10, DppV, and beta-glucosidase were found in guinea pig infections with *Arthroderma benhamiae* (12). Metalloendoproteinases (MEP2 and MEP4) and subtilisins (Sub5, Sub2, Sub3, Sub1, and Sub4) were identified previously in microarray and expression data of *T. rubrum* when grown on media containing skin proteins or skin sections as well (13, 14). As indicated, among these enzymes, proteases have been predominant and garnered the most attention. However, focus on specific protease members (viz., Sub6, Sub7, Sub8, and so on) presents an imperfect view as other secreted proteins (viz., glucan 1,3-beta-glucosidase and beta-glucosidase) that also act as key virulence factors or allergens are often ignored or neglected. Moreover, the expression of individual subtilisin and metalloproteinase appears to be context dependent, depending on pathogen and/or growth conditions. Hence, an alternate function-based categorization aided by a multi-omics approach utilizing liquid chromatography-tandem mass spectrometry (LC-MS) and RNA-seq is presented here that provides a robust holistic view of the critical pathways associated with key virulence factors.

**Fig 1:**
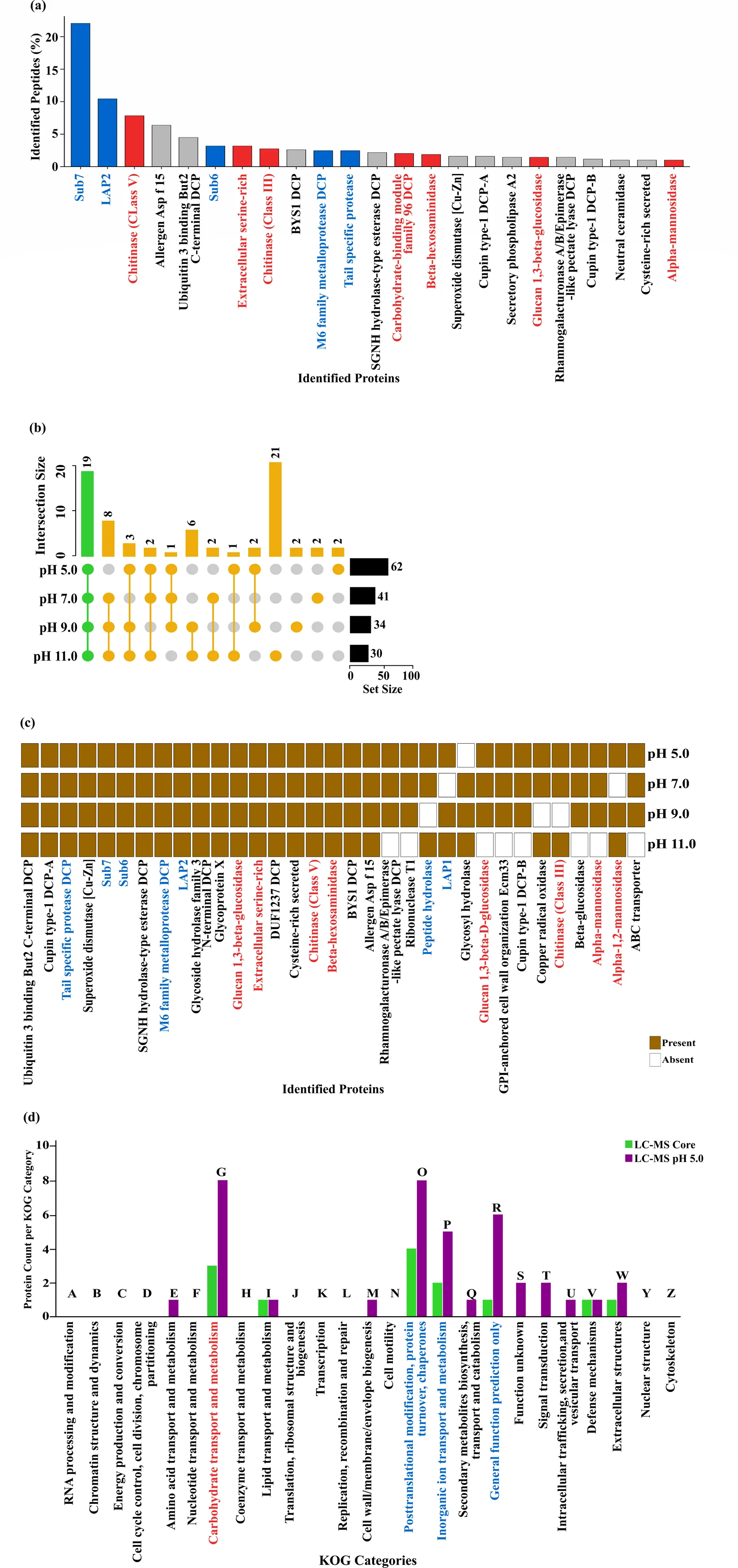
Secretome analysis of *T. rubrum.* (**a**) Proteins identified at pH 5.0 by proteomics with at least 1% of total peptides (i.e., a minimum of seven peptides [Table S1]) are represented for clarity. Proteases and carbohydrate-metabolizing enzymes are indicated in blue and red colors, respectively, across all panels. (**b**) An UpSet plot showing the distribution of identified proteins across four different pH conditions. Each bar represents the total number of proteins detected under the indicated pH conditions. Proteins detected in all four pH conditions are designated as core proteins (green: detection in all indicated pH, gold: detected at indicated pH, and gray: not detected). (**c**) List of proteins detected at least three of the four pH conditions. (**d**) Functional categorization (KOG) of identified proteins in pH 5.0 and the core protein set. Protein annotation “Domain-containing proteins” has been abbreviated as DCP.

The secretome of *T. rubrum* IGIB-SBL-CI1 was first examined at acidic pH, i.e., pH 5.0. The dermatophyte was grown on 0.2% soy protein media, and the repertoire of secreted proteins was examined using LC-MS. A total of 689 peptides representing 62 distinct secreted proteins were identified, at a false discovery rate (FDR) < 0.05% with 95% sequence similarity, and at least one unique peptide per protein (Table S1). The most abundant peptides in the secretome at pH 5.0 were captured for Sub7 (TERG_05699), belonging to the S8 family of serine peptidases, and LAP2 (TERG_08405), belonging to the M28 family of metallopeptidases (Fig. 1a). Sub6 (TERG_02990), M6 metalloprotease (TERG_01805), and tail protease (TERG_08195) were the other major proteases in the secretome (Fig. 1a; Table S1). In addition, Sub3 (TERG_03815), Sub9 (TERG_08008), LAP1 (TERG_05652), peptide hydrolase (TERG_03681), and prolidase (TERG_07085) were also identified (Table S1).

There are 16 members of S8 subtilases in *T. rubrum* (15), out of which only Sub3, Sub6, Sub7, and Sub9 were captured in LC-MS, indicating a temporo-spatial production of subtilisins is likely and other members of this family of proteases could be expressed and secreted at different growth stages. The presence of multiple secreted peptidases identified in the extracellular milieu, however, suggests that the fungus is actively degrading environmental proteins into peptides and amino acids. Several previous studies indicate varying protein profiles of examined secretomes in different dermatophyte species, limiting assessment of key enzymes across different species (Table S2). Instead, given the similar environment that the dermatophytes encounter, i.e., limited nutrients on the terminally differentiated keratinocyte layer during infection, a functional category-based examination (e.g., KOG or GO) can give a generalized view for comparison of key functional machineries and pathways associated with pathogenicity and utilization of these limited proteins on the host. The identified endo- and exo-proteases in the secretome at pH 5.0 were classified into various functional groups of KOG classification and found to belong to categories O, P, and R (Fig. 1d; Table S3).

Secreted endo- and exo-proteases are a key family of enzymes that help dermatophytes in establishing infection in the acidic environment of the host skin (Fig. S1). During infection, however, keratin degradation and metabolism lead to the accumulation of tripeptides, dipeptides, and amino acids (Fig. S1). The metabolism of amino acids results in the secretion of ammonia and shifts the ambient pH from acidic to alkaline (9, 16). Hence, in addition to the skin-mimic pH condition (pH 5.0), the secretome was also analyzed at neutral (pH 7.0) and alkaline (pH 9.0 and pH 11.0) pH conditions to capture the core protein subset of *T. rubrum* (Fig. 1b).

A total of 1,732 peptides corresponding to 71 distinct proteins were identified at all four pH conditions combined. Distribution of proteins across the different pH conditions identified 19 core proteins in all four pH and confirmed KOG categories O, P, and R for posttranslational modifications, proteases, and ion transport (representing protein turnover), and KOG category G (representing carbohydrate-metabolizing enzymes) as the core protein functional categories required for dermatophyte growth (Fig. 1b through d).

Members of KOG category G were next analyzed in the secretome. Chitinases (TERG_00216 and TERG_02705) and the extracellular serine-rich protein (TERG_05063) were the major proteins of KOG category G, i.e., carbohydrate-metabolizing enzymes with a high number of peptides in the secretome (Fig. 1a; Table S1). Beta-hexosaminidase, glucan endo-1,3-beta-D-glucosidase, and alpha-1,2-mannosidase were the other predominant enzymes in the secretome required for synergistic adaptive metabolism for breakdown of complex substrates that contain both protein and carbohydrates in the soy media (Fig. 1a). These KOG category G enzymes (1,3-beta-glucanosyltransferase, chitinase, and alpha-1,2-mannosidase) have previously been shown to be upregulated in keratin media, *in vitro* and *ex vivo* growth conditions, and associated with cell wall remodeling and fungal virulence (10, 17).

In addition to proteases and carbohydrate-metabolizing enzymes, fungi-specific proteins, namely BYS1 domain-containing protein involved in cell wall morphogenesis and the allergen Asp f15 protein known to induce allergic reactions in humans, were the other captured proteins in the LC-MS experiment.

**Fig 2:**
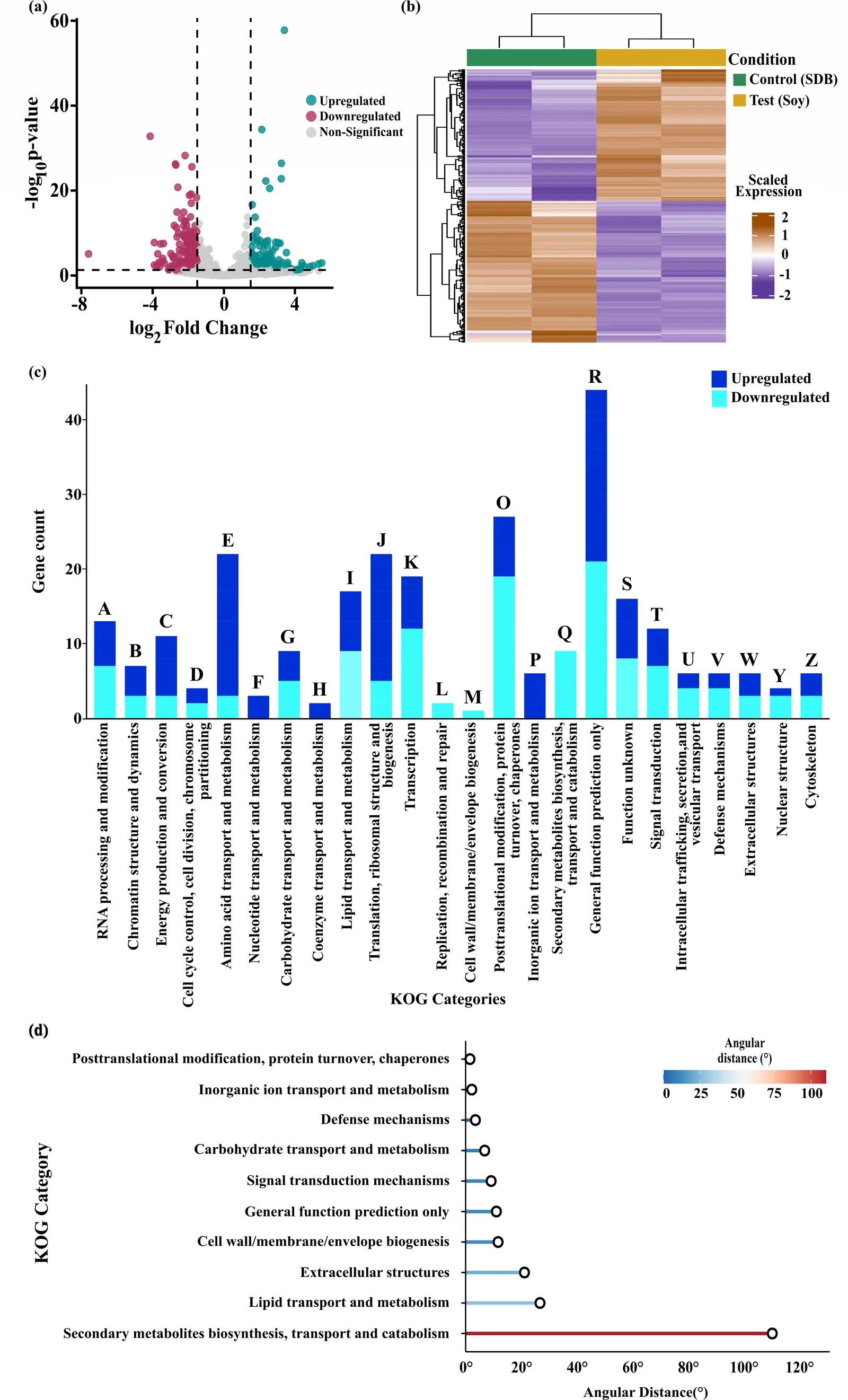
Transcriptome profile and correlation analysis of multi-omics data. (**a**) Volcano plot, (**b**) heatmap, and (**c**) functional categorization (KOG) for DEGs in soy protein media. (**d**) Correlation analysis showing RNA-protein concordance per pathway.

To obtain an overview of key components expressed at pH 5.0 in protein-rich soy media, transcriptome profiling by long-read Oxford Nanopore Technologies sequencing of *T. rubrum* was carried out, followed by correlation analysis of the proteomics and transcriptomics data. Transcriptional profiling identified differentially expressed genes (DEGs) in the protein-rich soy media vs growth in Sabouraud’s dextrose broth (SDB), using the DESeq2 R package. Overall, 388 significantly altered DEGs were identified based on adjusted *P*-values < 0.05 and log2 fold change (±1.5) thresholds (Fig. 2a and b; Fig. S2; Table S4). The transcriptome results were validated using qRT-PCR for a few selected genes before further analysis and showed a highly similar trend of change in expression levels (upregulation or downregulation) for the tested genes (Table S5). A significant modulation of genes involved in protein metabolism, including those encoding proteases, amino acid transporters, and enzymes of amino acid biosynthesis and catabolism (i.e., KOG categories E, O, and R), was identified for the transcriptome (Fig. 2c; Table S4). The transcriptional activation of these pathways related to protein digestion and assimilation indicated a coordinated regulatory response to protein-rich conditions, suggesting that *T. rubrum* not only produced extracellular enzymes to liberate amino acids but also activated the intracellular machinery necessary for their uptake and utilization (Fig. 1d and 2c). In contrast, KOG-based functional enrichment in SDB revealed genes associated with translation, ribosomal structure, and biogenesis (KOG category J) as the most upregulated, suggesting that the cellular machinery is diverted towards growth (Fig. S3c) during the availability of complete nutrients.

A pathway-level integration of transcriptomics and proteomics data was next assessed through a vector-based similarity approach. Two vectors: one representing proteomic enrichment (LC-MS; i.e., peptide proportions normalized by protein length and pathway gene count), and the other representing transcriptomic pathway enrichment scores (RNA-seq; ssGSEA) were constructed for each KOG pathway (Fig. S4) (18). To quantify concordance, a cosine similarity between the RNA and protein vectors was computed and assessed based on the cosine angle, i.e., 0° indicating perfect alignment, 90° indicating no correlation, and 180° indicating negative correlation. High concordance was identified for KOG categories O (posttranslational modification, protein turnover, and chaperones) and P (inorganic ion transport and metabolism) in the multi-omics correlation analysis (Fig. 2d). Category G (carbohydrate transport and metabolism) identified in the secretome also shows concordance with the transcriptome data, as per cosine similarity analysis. Interestingly, KOG category V (defense mechanisms) also exhibited significant concordance in the multi-omics correlation analysis.

Together, these results link transcriptional regulation to enzymatic function of key functional categories and virulence factors, namely, protein turnover (for invasion, pH modulation, and nutrient acquisition) and carbohydrate-metabolizing enzymes (cell-wall remodeling, adhesion, and invasion) during infection. The interplay between protein turnover or proteolysis, carbohydrate metabolism, and defense pathways (Fig. 2d) highlights the complexity of nutrient adaptation and response to environmental cues across filamentous dermatophytic fungi and will aid the identification of key pathways and virulence factors required during growth and infection.

## Data Availability

The proteomics data generated in this study has been deposited at the Open Science Framework (OSF; https://osf.io/gyf48/?view_only=e7c6f872c1be48a5a5cc643621f80451). RNA-sequencing data is deposited at National Center for Biotechnology Information with BioProject accession number PRJNA1336754.

## SUPPLEMENTAL MATERIAL

Supplemental Material spectrum.03483-25-s0001.docx Supplemental methods, Fig. S1 to S4, and Tables S1 to S5.

ASM does not own the copyrights to Supplemental Material that may be linked to, or accessed through, an article. The authors have granted ASM a non-exclusive, world-wide license to publish the Supplemental Material files. Please contact the corresponding author directly for reuse.
