# Supplementary material for: An integrated proteomics and transcriptomics analysis highlights concordance between protein turnover and carbohydrate transport and metabolism as key functional categories during the growth of Trichophyton rubrum
Source: Microbiol Spectr. 2026 Aug 4;14(9):e03483-25. doi: 10.1128/spectrum.03483-25 (PMC13532276; doi:10.1128/spectrum.03483-25)
Supplement: Supplementary file 1 [file spectrum.03483-25-s0001.docx]

**Supplementary Data**

**An integrated proteomics and transcriptomics analysis highlights concordance between protein turnover and carbohydrate transport and metabolism as key functional categories during growth of *Trichophyton rubrum***

Himani Singh^1,2^, Chitra Latka^1,2^, Rahul Chakraborty^1,2^, Leepakshi Dhingra^1^, Sherry Bhalla^1,2^ and Bhupesh Taneja^1,2*^

**List of Supplementary Materials and Methods**

(a) Fungal strain and culture conditions
(b) Protein extraction and trypsin digestion
(c) HPLC fractionation and LC-MS analysis
(d) RNA isolation, cDNA synthesis and sequencing
(e) Data processing and sequencing analysis
(f) Pathway-level integration of transcriptomics and proteomics
(g) qRT-PCR validation of RNA-seq

**List of Supplementary Figures:**

**Figure S1:** Illustrated steps during pathogenesis of dermatophytosis.

**Figure S2:** Correlation between biological duplicates of samples for transcriptomic analysis.

**Figure S3:** Transcriptomic profile of *T. rubrum* on rich media (SDB).

**Figure S4:** Score calculation for multi-omics data, to estimate concordance.

**List of Supplementary Tables:**

**Table S1:** List of secreted proteins at different pH levels.

**Table S2**: List of major secreted proteins identified across various studies.

**Table S3:** KOG categories for identified proteins.

**Table S4:** Log_2_FC values for genes modulated in the transcriptomic profile of *T. rubrum* on soy protein media.

**Table S5:** Validation of transcriptome using qRT-PCR

**Supplementary Methods**

(a) **Fungal strain and culture conditions**

*Trichophyton rubrum* IGIB-SBL-CI1, isolated previously from a patient with onychomycosis (Latka et al. 2015), was maintained on Sabouraud’s dextrose agar at 30 ºC. For secretome analysis, *T. rubrum* was grown without shaking on 0.2% soy protein media at 30 ºC for 30 days at different pH levels (pH 5.0, pH 7.0, pH 9.0, and pH 11.0). For transcriptome analysis, 3x10³ spores/ml were cultured in 0.2% soy protein media for the test and in Sabouraud’s dextrose broth (for control) at 30˚C for 7 and 14 days in two replicates.

(b) **Protein extraction and trypsin digestion**

The media from the grown cultures was collected. The collected supernatant, containing extracellularly secreted proteins, was filtered through two layers of muslin cloth. The secreted proteins were precipitated overnight with 15% v/v trichloroacetic acid at 4 °C. The precipitated protein was separated by centrifuging at 15000g, 4 °C for an hour. The protein pellet was washed thrice with 100% cold acetone and then allowed to dry at room temperature (RT). Proteins were then dissolved in 8M urea (overnight), and the soluble fraction was removed after centrifugation at 15000g for one hour. Denatured proteins were then dialysed against 20 mM Tris-HCl (pH 8.0), and protein concentration was determined using the bradford method. Proteins were reduced with 10mM DTT followed by alkylation with 20mM iodoacetamide. Proteins were digested with trypsin (Promega) at 37 °C overnight and lyophilized in a speed-vac vacuum concentrator in an aqueous mode for further use.

(c) **HPLC fractionation and LC-MS analysis**

Trypsin-digested peptides were reconstituted in 25µl of LC-MS grade water containing 0.1% formic acid. They were then run on a quadrupole-TOF hybrid mass spectrometer (TripleTOF6600, Sciex, USA) coupled to a nano-LC system (Eksigent NanoLC-400). 2 µl of the sample was injected and loaded onto a reverse-phase peptide ChromoLC trap (200µm x 0.5 mm) column. Peptides were separated using a C18 column (75 µm x 150 mm, Eksigent). The samples were run using a gradient method with buffer A (2% LC-MS grade acetonitrile + 97.9% LC-MS water + 0.1% formic acid) and buffer B (99.9% acetonitrile + 0.1% formic acid). Data was acquired with a NanoSpray source installed in the TripleTOF 6600 System using a nebulizing gas at 20 psi, a curtain gas at 25 psi, an ion spray voltage of 2000 V, and a heater interface temperature of 75 ˚C. Information-dependent acquisition (IDA) mode was set up with a TOF/MS survey scan (350–1600 m/z) with an accumulation time of 250 ms. For fragmentation, a maximum of ten precursor ions per cycle were selected with each MS/MS spectrum (200–1800 m/z) accumulated for 70 ms with a total cycle time of approximately 2.05 seconds. Parent ions with a charge state from +2 to +5 and an abundance of more than 150 cps were selected for MS/MS fragmentation. Once an ion had been fragmented by MS/MS, its mass and isotopes were excluded for a period of 3 s. The wiff files generated from Triple-TOF 6600 were analyzed using the Protein Pilot v4.0 to identify the desired protein against UniProt's *Trichophyton rubrum* protein database. MycoCosm (Grigoriev et al. 2014) (<https://mycocosm.jgi.doe.gov/mycocosm>) was used for providing KOG annotations to all the identified proteins. KOG categories were identified for pooled data containing peptide information from both replicates.

(d) **RNA isolation, cDNA synthesis and sequencing**

Grown mycelia were filtered through muslin cloth and powdered using liquid nitrogen. Trizol (1ml/60mg powdered mycelia) was added at RT for 10-15 min. Chloroform (0.2 mL/mL of sample) was then added, mixed thoroughly and incubated for 10 min, followed by centrifugation at 15000g for 10 min at RT to separate aqueous and organic phases. The aqueous phase, containing RNA, was carefully transferred to a fresh microcentrifuge tube, and RNA was precipitated with isopropanol. Following centrifugation, the RNA pellet was washed with 75% ethanol, air-dried briefly, and resuspended in RNase-free water.

rRNA was depleted from the total RNA using the QIAseq FastSelect-rRNA yeast kit. Following depletion, the RNA was purified using RNA Clean XP beads. Purified mRNA was used for the double-stranded cDNA synthesis kit using NEB first and second strand synthesis kit, as per manufacturer instructions. The resulting cDNA was purified and quantified before library preparation and sequencing.

Prepared ds-cDNA was used for library preparation, using native barcoding kit, SQK-NBD114 (Oxford Nanopore Technologies, ONT). The double-stranded cDNA was first subjected to end-repair and dA-tailing, followed by purification with AMPure XP beads. Native barcodes were then ligated to the prepared ends using the barcode ligation mix, samples were pooled and subjected to another round of bead purification. The final library was quantified and prepared for loading onto a MinION flow cell. All steps were carried out at ONT-recommended temperatures and durations to ensure optimal ligation and sequencing efficiency.

(e) **Data processing and sequencing analysis**

After the sequencing run was completed, raw signal data (FAST5 format) were basecalled in high accuracy mode using Guppy Basecaller (Wick et al., 2019). Following basecalling, demultiplexing and barcode removal were performed using Guppy’s in-built barcode parameters. The ribosomal RNA (rRNA) sequences were removed from the obtained FastQ files using RiboDetector (Deng et al., 2022). The obtained non-rRNA reads were next analysed for downstream transcriptomic analysis.

Obtained reads were aligned to the *T. rubrum* reference genome using STAR Aligner (Dobin et al., 2013). Aligned reads were then counted for respective genes using HTseq-count and subjected to differential gene expression analysis using DESeq2 of R package. Normalised data was utilized to create a correlation plot between the biological duplicates. Genes exhibiting a p-value cutoff <0.05 and a log_2_FC >±1.5 were considered statistically significant and defined as Differentially expressed genes (DEGs). DEGs were categorised into KOG Classes to identify regulated pathways.

(f) **Pathway-level integration of transcriptomics and proteomics**

To compare RNA-seq and proteomics data at the pathway level, raw counts were generated using HTSeq-count for RNA-seq data, while peptide-level quantification was carried out for the LC-MS data. Gene-level counts were combined into a count matrix, and variance-stabilizing transformation (VST) was applied using DESeq2. Single-sample Gene Set Enrichment Analysis (ssGSEA) was used to compute pathway activity scores for each sample using a custom KOG pathway GMT file, implemented with GSVA.

**Proteomics normalization and pathway-level summarization:** LC-MS peptide intensities were normalized by gene length to account for gene size bias:

$$Normalized Peptides_{g}=\frac{\% Peptides Detected_{g}}{Gene Length_{g}}$$

Normalized peptide values were summed across all genes in each KOG pathway P to generate pathway-level protein abundance:

$Abundance_{P}=\sum_{g \in P} Normalized Peptides_{g}$

To focus on secretory proteins, the pathway-level abundance was further divided by the number of secretory proteins in the pathway:

$$Secretory Normalized Abundance_{P}=\frac{Number of Secretory Proteins in P}{Abundance_{P}}$$

**Integration of RNA-seq and proteomics data:** Pathways common to both RNA ssGSEA scores and protein abundances were retained. Cosine similarity between RNA and protein pathway profiles was computed for each pathway:

$$Cosine Similarity_{P}=\frac{\Sigma sX_{P,S}Y_{P,S}}{\sqrt{\Sigma_{s}X_{P,S}^{2}\sqrt{\Sigma_{s}Y_{P,S}^{2}}}}$$

Where $X_{P,S}$ and $Y_{P,S}$ are the RNA and protein values for the pathway $P$in sample $S$. Angular distance in degrees was then calculated as:

$$Angular Distance_{P}=arccos \left( Cosine Similarity_{P} \right) \times\frac{180}{\pi}$$

Angular distance was then calculated in degrees using the inverse cosine of the similarity score to facilitate visualization. Smaller angles indicate higher concordance between RNA and protein measurements, whereas larger angles indicate greater divergence between the two datasets.

**(g) qRT-PCR validation of RNA-seq data**

Total RNA was extracted from the fungal samples using TRIzol reagent (Invitogen). Fungal biomass was homogenized in liquid nitrogen, followed by phase separation with chloroform and RNA precipitation using isopropanol. The isolated RNA was treated with DNase I to eliminate residual genomic DNA contamination. RNA quality and concentration were assessed prior to cDNA synthesis. 1 µg of RNA was reverse transcribed into cDNA using the SuperScrip III First-Strand Synthesis kit (Invitrogen) following the manufacturer's protocol. Quantitative real-time PCR (qRT-PCR) was performed using SYBR Green Master Mix from Takara Bio. Gene-specific primers were used as follows:

| **Gene** | **Primers Sequences** |
| --- | --- |
| **Subtilisin-like protease 4** (TERG_01617) (Sub4) | **FP:** GACTCCAAGGTCTACGTT  **RP:** TGTCGGTGAGAGACGCGT |
| **Subtilisin-like protease 3** (TERG_03815) (Sub3) | **FP:** CCTCAAAGATGACAAGGTCAACTAG  **RP:** AGGAGCCTTGTGGGAGACT |
| **Leucine Aminopeptidase 1** (TERG_05652) (LAP1) | **FP:** AAGAAGGTCAAGGGCATGCTCA  **RP:** ATTGAGCAGTACTTGGTGATGACC |
| **Hsp90-like protein (TERG_06963)** | **FP:** ACCGTGCTGCCCTTGCT  **RP:** GTGATCTCGTCGCCAGACTTG |
| **Siderochrome-iron transporter Sit1** (TERG_03174) | **FP:** CGGCGTCAAGGCGTTCTGT  **RP:** GGAGCGAGGTCACGTCCG |
| **Sterol 24-C-methyltransferase** (TERG_03102) | **FP:** ACTTCATGGAGATGGATTTCC  **RP:** CGTCATACTTGTCCGTCATTA |
| **SAM-dependent methyltransferase Erg6/SMT-type domain-containing protein** (TERG_05431) | **FP:** ATAATGTCTGGTGAGAGTAGC  **RP:** CCTTTAACAAGGTCCTGCA |
| **PacC** (RT-Control) | **FP:** TCCCAGCAGCCCCAAC  **RP:** ATGTGGGAGGTGATGTGGT |
| **Beta-actin** (Housekeeping gene) | **FP:** GAATCTCCCCCCTCATGCTT  **RP:** GCCTGGGCTTCCGACACT |

The beta-actin gene was used as the internal reference for normalization after confirming its stable expression across all experimental conditions. Relative gene expression was calculated using the 2^-ΔΔCt^ method. To facilitate comparison with RNA-seq differential expression results, the fold change values were transformed to log_2_ fold change (log_2_FC) by calculating log_2_(2^-ΔΔCt^). The expression profiles of selected genes obtained by qRT-PCR were compared with the corresponding RNA-seq log_2_FC values to validate the transcriptomic data.

**Figure S1: Illustrated steps during pathogenesis of dermatophytosis.** Dermatophytosis begins with contact of arthroconidia (spores) to the outermost layer of the skin (the stratum corneum). The spores then adhere to human skin using carbohydrate-hydrolyzing enzymes, including chitinases, glucanases, and mannosidases, that interact with host glycans, promoting adhesion to the skin surface and enabling deeper penetration. Once adherence is established, the spores germinate and form hyphae, which start to grow outward and penetrate deeper layers of the stratum corneum. As the fungus invades keratinized tissue, it secretes a repertoire of both endo-proteases and exo-proteases. Keratin is first denatured by disulfide reductases through cleavage of disulfide bonds, followed by breakdown of keratin protein into short peptides by endopeptidases (such as subtilisins and metalloproteinases). These peptides are further broken down into oligopeptides by exopeptidases such as LAPs and DppIV, and finally into di-, tri-peptides and amino acids so that the fungus can assimilate them for growth. Further metabolism of some amino acids, viz., glycine, results in secretion of ammonia resulting in the alkalinization of the skin and subsequent activation of alkaline keratinases and proteases for adaptation at the alkaline pH conditions.

**Figure S2: Correlation between biological duplicates of samples for transcriptomic analysis.**

**Figure S3: Transcriptome profile of *T. rubrum* on rich media (SDB). (a)**Volcano plot, **(b)** heatmap and **(c)** functional categorization (KOG) for DEGs in rich media (SDB).

**Figure S4: Score calculation for multi-omics data, to estimate concordance. (a)** Protein abundance per pathway from the secretome (LC-MS), **(b)** ssGSEA score for Transcriptomic profile in protein-rich media (RNA-Seq) and **(c)** Calculated cosine angle and similarity values indicating concordance of functional KOG pathways using LC-MS and RNA-seq data. KOG categories O, P, V and G with cosine similarity score of >0.99 were taken up as high concordance pathways as described in the main text.

**Table S1: List of secreted proteins at different pH levels.** The complete list of secreted proteins at the indicated pH (i.e. pH 5.0, pH 7.0, pH 9.0 and pH 11.0) is presented. The top proteins with at least 1% of total peptides (i.e. minimum 7 identified peptides for a total of 689 peptides) for pH 5.0 are highlighted in bold.

| **Secreted Proteins at pH 5.0** | | |
| --- | --- | --- |
|  |  |  |
| **Gene ID** | **Protein Name** | **No. of peptides** |
| **TERG_05699** | **Subtilisin-like protease 7 (Sub7)** | **152** |
| **TERG_08405** | **Leucine aminopeptidase 2 (LAP2)** | **72** |
| **TERG_00216** | **Chitinase (Class V)** | **54** |
| **TERG_02993** | **Allergen Asp f 15** | **44** |
| **TERG_06780** | **Ubiquitin 3 binding protein But2 C-terminal domain-containing protein** | **31** |
| **TERG_02990** | **Subtilisin-like protease 6 (Sub6)** | **22** |
| **TERG_05063** | **Extracellular serine-rich protein** | **22** |
| **TERG_02705** | **Chitinase (Class III)** | **19** |
| **TERG_00060** | **BYS1 domain-containing protein** | **18** |
| **TERG_01805** | **M6 family metalloprotease domain-containing protein** | **17** |
| **TERG_08195** | **Tail specific protease domain-containing protein** | **17** |
| **TERG_03459** | **SGNH hydrolase-type esterase domain-containing protein** | **15** |
| **TERG_03702** | **Carbohydrate-binding module family 96 domain-containing protein** | **14** |
| **TERG_04775** | **Beta-hexosaminidase** | **13** |
| **TERG_04335** | **Superoxide dismutase [Cu-Zn]** | **11** |
| **TERG_08180** | **Cupin type-1 domain-containing protein-A** | **11** |
| **TERG_00127** | **Secretory phospholipase A2** | **10** |
| **TERG_06242** | **Glucan 1,3-beta-glucosidase** | **10** |
| **TERG_06986** | **Rhamnogalacturonase A/B/Epimerase-like pectate lyase domain-containing protein** | **10** |
| **TERG_03492** | **Cupin-type I domain-containing protein-B** | **8** |
| **TERG_00899** | **Neutral ceramidase** | **7** |
| **TERG_04047** | **Cysteine-rich secreted protein** | **7** |
| **TERG_12680** | **Alpha-mannosidase** | **7** |
| TERG_00481 | Beta-glucosidase | 6 |
| TERG_01280 | IgE-binding protein | 6 |
| TERG_07641 | GPI-anchored cell wall organization protein Ecm33 | 6 |
| TERG_05627 | Secreted LysM effector LysM1 | 5 |
| TERG_06929 | Chitinase | 5 |
| TERG_06944 | Ribonuclease T1 | 5 |
| TERG_11886 | Copper radical oxidase | 5 |
| TERG_04806 | ABC transporter | 4 |
| TERG_04887 | Glucan endo-1,3-beta-D-glucosidase | 4 |
| TERG_05327 | DUF1237 domain-containing protein | 4 |
| ACT | Actin | 3 |
| TERG_01837 | Glycoside hydrolase family 3 N-terminal domain-containing protein | 3 |
| TERG_04747 | Phosphatidylglycerol/phosphatidylinositol transfer protein | 3 |
| TERG_06210 | Alkaline phosphatase | 3 |
| TERG_08225 | Glycoprotein X | 3 |
| TERG_08523 | Glycoside hydrolase family protein | 3 |
| A0A059K9M3 | Gamma-glutamyltransferase | 2 |
| TERG_00583 | Blue (type 1) copper domain-containing protein | 2 |
| TERG_02112 | Uncharacterized protein (TERG_02112) | 2 |
| TERG_02140 | BZIP transcription factor | 2 |
| TERG_03815 | Subtilisin-like protease 3 (Sub3) | 2 |
| TERG_07085 | Prolidase (Xaa-Pro /Aminoacylproline aminopeptidase) | 2 |
| TERG_07406 | Alpha-1,2-Mannosidase | 2 |
| A0A059JTE7 | FAD-linked oxidoreductase family | 1 |
| TERG_03624 | SUN domain-containing protein | 1 |
| TERG_03681 | Peptide hydrolase | 1 |
| TERG_05625 | Chitinase (Class V) | 1 |
| TERG_05652 | Leucine aminopeptidase 1 (LAP1) | 1 |
| TERG_07238 | Glucoamylase (EC 3.2.1.3) (1,4-alpha-D-glucan glucohydrolase) (Glucan 1,4-alpha-glucosidase) | 1 |
| TERG_07416 | Peroxin-7 | 1 |
| TERG_07490 | Secreted protein | 1 |
| TERG_07521 | Yos1-like protein | 1 |
| TERG_07757 | Peptidase S8/S53 domain-containing protein | 1 |
| TERG_07826 | Multifunctional tryptophan biosynthesis protein | 1 |
| TERG_07959 | Glucan 1,3-beta-glucosidase (Exo-1,3-beta-glucanase) | 1 |
| TERG_08008 | Subtilisin-like protease 9 (Sub9) | 1 |
| TERG_08011 | Metallocarboxypeptidase A-like protein | 1 |
| TERG_08419 | Mitochondrial ribosomal protein subunit S18 | 1 |
| TERG_11814 | Berberine/berberine-like domain-containing protein | 1 |

| **Secreted Proteins at pH 7.0** | | |
| --- | --- | --- |
|  |  |  |
| **Gene ID** | **Protein Name** | **No. of peptides** |
| TERG_05699 | Subtilisin-like protease 7 (Sub7) | 119 |
| TERG_08405 | Leucine aminopeptidase 2 (LAP2) | 54 |
| TERG_00216 | Chitinase (Class V) | 46 |
| TERG_04047 | Cysteine-rich secreted protein | 31 |
| TERG_02993 | Allergen Asp f 15 | 30 |
| TERG_06780 | Ubiquitin 3 binding protein But2 C-terminal domain-containing protein | 28 |
| TERG_05063 | Extracellular serine-rich protein | 21 |
| TERG_00060 | BYS1 domain-containing protein | 14 |
| TERG_01805 | M6 family metalloprotease domain-containing protein | 12 |
| TERG_02990 | Subtilisin-like protease 6 (Sub6) | 12 |
| TERG_08180 | Cupin type-1 domain-containing protein-A | 10 |
| TERG_03492 | Cupin-type I domain-containing protein-B | 9 |
| TERG_02705 | Chitinase (Class III) | 8 |
| TERG_02875 | Choline-phosphate cytidylyltransferase | 8 |
| TERG_03702 | Carbohydrate-binding module family 96 domain-containing protein | 8 |
| TERG_00127 | Secretory phospholipase A2 | 7 |
| TERG_03459 | SGNH hydrolase-type esterase domain-containing protein | 7 |
| TERG_06986 | Rhamnogalacturonase A/B/Epimerase-like pectate lyase domain-containing protein | 6 |
| TERG_04775 | Beta-hexosaminidase | 5 |
| TERG_07641 | GPI-anchored cell wall organization protein Ecm33 | 5 |
| TERG_08195 | Tail specific protease domain-containing protein | 5 |
| TERG_03815 | Subtilisin-like protease 3 (Sub3) | 4 |
| TERG_04335 | Superoxide dismutase [Cu-Zn] | 3 |
| TERG_04887 | Glucan endo-1,3-beta-D-glucosidase | 3 |
| TERG_05327 | DUF1237 domain-containing protein | 3 |
| TERG_06944 | Ribonuclease T1 | 3 |
| TERG_02112 | Uncharacterized protein (TERG_02112) | 2 |
| TERG_06242 | Glucan 1,3-beta-glucosidase | 2 |
| TERG_07959 | Glucan 1,3-beta-glucosidase (Exo-1,3-beta-glucanase) | 2 |
| TERG_12680 | Alpha-mannosidase | 2 |
| A0A028JV60 | Glycosyl hydrolase | 1 |
| A0A059JXK9 | FAD-dependent oxygenase family | 1 |
| TERG_00481 | Beta-glucosidase | 1 |
| TERG_01837 | Glycoside hydrolase family 3 N-terminal domain-containing protein | 1 |
| TERG_03681 | Peptide hydrolase | 1 |
| TERG_04806 | ABC transporter | 1 |
| TERG_05901 | Rad4 family protein | 1 |
| TERG_06210 | Alkaline phosphatase | 1 |
| TERG_08225 | Glycoprotein X | 1 |
| TERG_11886 | Copper radical oxidase | 1 |
| TERG_11887 | WSC domain-containing protein | 1 |

| **Secreted Proteins at pH 9.0** | | |
| --- | --- | --- |
|  |  |  |
| **Gene ID** | **Protein Name** | **No. of peptides** |
| TERG_05699 | Subtilisin-like protease 7 (Sub7) | 70 |
| TERG_00060 | BYS1 domain-containing protein | 53 |
| TERG_08405 | Leucine aminopeptidase 2 (LAP2) | 43 |
| TERG_06780 | Ubiquitin 3 binding protein But2 C-terminal domain-containing protein | 33 |
| TERG_00216 | Chitinase (Class V) | 30 |
| TERG_04047 | Cysteine-rich secreted protein | 18 |
| TERG_05063 | Extracellular serine-rich protein | 16 |
| TERG_06242 | Glucan 1,3-beta-glucosidase | 11 |
| TERG_02993 | Allergen Asp f 15 | 12 |
| TERG_04335 | Superoxide dismutase [Cu-Zn] | 7 |
| TERG_08180 | Cupin-type I domain-containing protein-A | 7 |
| TERG_02990 | Subtilisin-like protease 6 (Sub6) | 6 |
| TERG_01805 | M6 family metalloprotease domain-containing protein | 5 |
| TERG_08195 | Tail specific protease domain-containing protein | 5 |
| TERG_00481 | Beta-glucosidase | 4 |
| TERG_03459 | SGNH hydrolase-type esterase domain-containing protein | 4 |
| TERG_04775 | Beta-hexosaminidase | 4 |
| TERG_07641 | GPI-anchored cell wall organization protein Ecm33 | 4 |
| TERG_03492 | Cupin-type I domain-containing protein-B | 3 |
| TERG_06944 | Ribonuclease T1 | 3 |
| TERG_06986 | Rhamnogalacturonase A/B/Epimerase-like pectate lyase domain-containing protein | 3 |
| TERG_08204 | Carrier domain-containing protein | 3 |
| A0A028JV60 | Glycosyl hydrolase | 2 |
| TERG_07406 | Alpha-1,2-Mannosidase | 2 |
| TERG_00899 | Neutral ceramidase | 1 |
| TERG_01837 | Glycoside hydrolase family 3 N-terminal domain-containing protein | 1 |
| TERG_02665 | Non-specific serine/threonine protein kinase | 1 |
| TERG_04747 | Phosphatidylglycerol/phosphatidylinositol transfer protein | 1 |
| TERG_04806 | ABC transporter | 1 |
| TERG_04887 | Glucan endo-1,3-beta-D-glucosidase | 1 |
| TERG_05327 | DUF1237 domain-containing protein | 1 |
| TERG_05652 | Leucine aminopeptidase 1 (LAP1) | 1 |
| TERG_08225 | Glycoprotein X | 1 |
| TERG_12680 | Alpha-mannosidase | 1 |

| **Secreted Proteins at pH 11.0** | | |
| --- | --- | --- |
|  |  |  |
| **Gene ID** | **Protein Name** | **No. of peptides** |
| TERG_05699 | Subtilisin-like protease 7 (Sub7) | 48 |
| TERG_08405 | Leucine aminopeptidase 2 (LAP2) | 31 |
| TERG_00060 | BYS1 domain-containing protein | 15 |
| TERG_02993 | Allergen Asp f 15 | 12 |
| TERG_05063 | Extracellular serine-rich protein | 10 |
| TERG_00216 | Chitinase (Class V) | 9 |
| TERG_02990 | Subtilisin-like protease 6 (Sub6) | 9 |
| TERG_04047 | Cysteine-rich secreted protein | 8 |
| TERG_06242 | Glucan 1,3-beta-glucosidase | 8 |
| TERG_06780 | Ubiquitin 3 binding protein But2 C-terminal domain-containing protein | 7 |
| TERG_03459 | SGNH hydrolase-type esterase domain-containing protein | 6 |
| TERG_02705 | Chitinase (Class III) | 5 |
| TERG_01805 | M6 family metalloprotease domain-containing protein | 4 |
| TERG_04775 | Beta-hexosaminidase | 4 |
| TERG_05652 | Leucine aminopeptidase 1 (LAP1) | 4 |
| TERG_08180 | Cupin-type I domain-containing protein-A | 4 |
| TERG_04335 | Superoxide dismutase [Cu-Zn] | 3 |
| A0A028JV60 | Glycosyl hydrolase | 2 |
| TERG_07406 | Alpha-1,2-Mannosidase | 2 |
| TERG_08195 | Peptidase S41 family protein/Tail specific protease domain-containing protein | 2 |
| TERG_08195 | Tail specific protease domain-containing protein | 2 |
| A0A059JXK9 | FAD-dependent oxygenase family | 1 |
| TERG_01837 | Glycoside hydrolase family 3 N-terminal domain-containing protein | 1 |
| TERG_02140 | BZIP transcription factor | 1 |
| TERG_03681 | Peptide hydrolase | 1 |
| TERG_05149 | EF-hand domain-containing protein | 1 |
| TERG_05327 | DUF1237 domain-containing protein | 1 |
| TERG_05511 | Thymocyte nuclear protein 1 | 1 |
| TERG_08225 | Glycoprotein X | 1 |
| TERG_11886 | Copper radical oxidase | 1 |
| TERG_11887 | WSC domain-containing protein | 1 |

**Table S2: List of major secreted proteins identified across various studies.** Highly expressed and/or proteins with a high number of unique peptides reported across various studies are summarised.

| **S.No** | **Identified Proteins** | **Study** | **References** |
| --- | --- | --- | --- |
| 1 | Sub2, Sub6, Sub7, MEP3, MEP4 | Identification of Proteins by LC-MS in soy media from *T. tonsurans, T. rubrum, T. equinum and T. violaceum*. | Giddey et al. (2007) |
| 2 | LAP1, LAP2, Beta-glucosidase, MEP3, Sub3, Sub4 | Identification of secreted proteins using 2D-PAGE and a shotgun mass spectrometry approach from *Trichophyton rubrum* and *Trichophyton violaceum*. | Giddey et al. (2007) |
| 3 | Sub3, Sub4, and MEP4 | *Trichophyton rubrum* expression was checked on keratin, collagen, elastin and human skin sections. | Leng et al. (2009) |
| 4 | Sub1, Sub2, Sub3, Sub4, Sub5, MEP2 and MEP4 | *T. rubrum* secreted proteases gene expressions from human nail and skin stratum corneum keratin as the growth medium. | Chen et al. (2010) |
| 5 | *M. canis*: SedB, SedD, Pep1 (pH4);  LAP1, Sub3, McpB, MEP4, DppIV (pH7) | Identified secreted Proteins in soy media of *M. canis* and *A. benhamiae* using LC-MS. | Sriranganadane et al. (2011) |
|  | *A. benhamiae:* DppV, Pep1, Sub3, SedB (pH4);  LAP2, S41, MEP4, Sub7 (pH7) |  |  |
| 6 | Sub6, Sub7, LAP2, DppV, Glucan 1,3-beta-glucosidase, Beta-glucosidase | Proteins identified by LC-MS/MS in onchomycosis patient infected with *T. rubrum*. | Méhul et al (2016) |
| 7 | Sub6, Sub7, Sub8, and Sub10, DppV, and the Beta- glucosidase | Secreted proteases and other allergen proteins during infection by *Arthroderma benhamiae* in guinea pigs. | Tran et al. (2016) |
| 8 | Sub1, Sub3, LAP1 and LAP2 | *T. rubrum* gene expression modulation on keratin and elastin substrates. | Bitencourt et al. (2016) |
| 9 | Sub7, Sub6, LAP2, Chitinase, Beta-hexosaminidase, Glucan 1,3-beta-glucosidase | Secreted Proteins Identified in *T. rubrum* using LC-MS in soy media at acidic pH. | This study |

**Table S3: KOG categories for identified proteins.** KOG category-based clustering of the complete protein list identified in the secretome.

| **KOG Category** | | **Protein Name (TERG_ID)** |
| --- | --- | --- |
| E | Amino acid transport and metabolism | Multifunctional tryptophan biosynthesis protein (TERG_07826) |
| G | Carbohydrate transport and metabolism | Chitinase (Class V) (TERG_00216) |
|  |  | Beta-hexosaminidase (TERG_04775) |
|  |  | Glucan endo-1,3-beta-D-glucosidase (TERG_04887) |
|  |  | Extracellular serine-rich protein (TERG_05063) |
|  |  | Chitinase (Class V) (TERG_05625) |
|  |  | Secreted LysM effector LysM1 (TERG_05627) |
|  |  | Chitinase (TERG_06929) |
|  |  | Alpha-1,2-Mannosidase (TERG_07406) |
| I | Lipid transport and metabolism | SGNH hydrolase-type esterase domain-containing protein (TERG_03459) |
| M | Cell wall/membrane/envelope biogenesis | Chitinase (Class III) (TERG_02705) |
| O | Posttranslational modification, protein turnover, chaperones | Subtilisin-like protease 6 (Sub6, TERG_02990) |
|  |  | Peptide hydrolase (TERG_03681) |
|  |  | Subtilisin-like protease 3 (Sub3, TERG_03815) |
|  |  | Extracellular serine-rich protein (TERG_05063) |
|  |  | Leucine aminopeptidase 1 (LAP1, TERG_05652) |
|  |  | Subtilisin-like protease 7 (Sub7, TERG_05699) |
|  |  | Subtilisin-like protease 9 (Sub9, TERG_08008) |
|  |  | Leucine aminopeptidase 2 (LAP2, TERG_08405) |
| P | Inorganic ion transport and metabolism | Peptide hydrolase (TERG_03681) |
|  |  | Superoxide dismutase [Cu-Zn] (TERG_04335) |
|  |  | Leucine aminopeptidase 1 (LAP1, TERG_05652) |
|  |  | Alkaline phosphatase (TERG_06210) |
|  |  | Leucine aminopeptidase 2 (LAP2, TERG_08405) |
| Q | Secondary metabolites biosynthesis, transport and catabolism | ABC transporter (TERG_04806) |
| R | General function prediction only | Peptide hydrolase (TERG_03681) |
|  |  | Phosphatidylglycerol/phosphatidylinositol transfer protein (TERG_04747) |
|  |  | Leucine aminopeptidase 1 (LAP1, TERG_05652) |
|  |  | Prolidase (Xaa-Pro /Aminoacylproline aminopeptidase) (TERG_07085) |
|  |  | Peptidase S8/S53 domain-containing protein (TERG_07757) |
|  |  | Leucine aminopeptidase 2 (LAP2, TERG_08405) |
| S | Function unknown | Yos1-like protein (TERG_07521) |
|  |  | Metallocarboxypeptidase A-like protein (TERG_08011) |
| T | Signal transduction mechanisms | Rhamnogalacturonase A/B/Epimerase-like pectate lyase domain-containing protein (TERG_06986) |
|  |  | Neutral ceramidase (TERG_00899) |
| U | Intracellular trafficking, secretion, and vesicular transport | Peroxin-7 (TERG_07416) |
| V | Defense mechanisms | Glycoprotein X (TERG_08225) |
| W | Extracellular structures | Glycoprotein X (TERG_08225) |
|  |  | SUN domain-containing protein (TERG_03624) |

**Table S4: Log_2_FC values for genes modulated in the transcriptomic profile of *T. rubrum* on soy protein media.**

| **KOG Category** | | **Protein Name (TERG_ID)** | **Log₂Fold Change** |
| --- | --- | --- | --- |
| A | RNA processing and modification | RRM domain-containing protein (TERG_00959) | -2.182238159 |
|  |  | H/ACA ribonucleoprotein complex subunit 2 (TERG_01368) | 3.564676536 |
|  |  | Uncharacterized protein (TERG_01597) | 1.671318993 |
|  |  | Annexin ANXC4 (TERG_02375) | -1.692718134 |
|  |  | FHA domain-containing protein (TERG_03619) | -1.959590885 |
|  |  | WW domain-containing protein (TERG_04235) | 2.070310337 |
|  |  | Activating signal cointegrator 1 complex subunit 3 (TERG_05471) | -2.495853299 |
|  |  | Uncharacterized protein (TERG_05703) | 1.681340755 |
|  |  | RRM domain-containing protein (TERG_06137) | 1.804681914 |
|  |  | Ataxin-2 C-terminal domain-containing protein (TERG_07624) | -1.571080198 |
|  |  | Polynucleotide adenylyltransferase (TERG_08045) | -2.028160882 |
|  |  | Protein kinase domain-containing protein (TERG_08278) | -2.022256549 |
|  |  | Ribosome assembly factor mrt4 (TERG_08497) | 3.040361996 |
| B | Chromatin structure and dynamics | Gfd2/YDR514C-like C-terminal domain-containing protein (TERG_00836) | 1.878209512 |
|  |  | Histone acetyltransferase type B subunit 2 (TERG_04518) | 1.569051691 |
|  |  | SET domain-containing protein (TERG_06122) | 2.292570361 |
|  |  | Uncharacterized protein (TERG_06740) | -2.09168514 |
|  |  | DUF7514 domain-containing protein (TERG_06875) | -1.699357749 |
|  |  | Deacetylase sirtuin-type domain-containing protein (TERG_07330) | -2.468492528 |
|  |  | CFEM domain-containing protein (TERG_08286) | 1.752793583 |
| C | Energy production and conversion | Dihydrolipoyl dehydrogenase (TERG_02599) | 3.014468444 |
|  |  | FAD-binding domain-containing protein (TERG_03218) | -1.635904572 |
|  |  | Malate dehydrogenase (TERG_03762) | 2.181943046 |
|  |  | Citrate synthase (TERG_04125) | 1.868375427 |
|  |  | ATP synthase subunit beta (TERG_04145) | 1.690656005 |
|  |  | Mitochondrial phosphate carrier protein (TERG_04714) | 2.333662641 |
|  |  | fumarate reductase (NADH) (TERG_05579) | -2.21709211 |
|  |  | ATP synthase subunit gamma (TERG_06183) | 2.075926482 |
|  |  | Homoaconitate hydratase (TERG_06569) | 2.297647835 |
|  |  | Nitrate reductase (TERG_07816) | -1.956407913 |
|  |  | ATP citrate synthase (TERG_08288) | 1.602554843 |
| D | Cell cycle control, cell division, chromosome partitioning | Uncharacterized protein (TERG_01627) | 1.860746847 |
|  |  | Protein kinase domain-containing protein (TERG_05544) | -2.21995463 |
|  |  | Metacaspase-1B (TERG_07319) | -1.903470346 |
|  |  | Translationally-controlled tumor protein homolog (TERG_08234) | 1.589660786 |
| E | Amino acid transport and metabolism | Uncharacterized protein (TERG_00215) | 1.806848218 |
|  |  | Pyruvate decarboxylase (TERG_00355) | 2.303563312 |
|  |  | Glutamate 5-kinase (TERG_00694) | 1.86538148 |
|  |  | Argininosuccinate synthase (TERG_00748) | 2.340627272 |
|  |  | Isocitrate dehydrogenase (TERG_01670) | 1.608737866 |
|  |  | Phosphoadenylyl-sulfate reductase (TERG_02449) | -1.638327922 |
|  |  | D-malate dehydrogenase (TERG_02773) | 2.136360071 |
|  |  | Aspartate aminotransferase (TERG_03187) | -2.10132313 |
|  |  | Amino acid permease/ SLC12A domain-containing protein (TERG_03886) | 1.648326458 |
|  |  | 3-methylcrotonyl-CoA carboxylase subunit alpha (TERG_04291) | 2.499363391 |
|  |  | methylcrotonoyl-CoA carboxylase (TERG_04294) | 1.907038444 |
|  |  | Amino acid permease (TERG_04356) | 1.871116552 |
|  |  | Asparagine synthase (TERG_04595) | 1.81300574 |
|  |  | Ornithine aminotransferase (TERG_04846) | 1.604154316 |
|  |  | Glycine cleavage system P protein (TERG_05419) | 1.826517865 |
|  |  | Cystathionine beta-lyase (TERG_05631) | 1.999842151 |
|  |  | Homoaconitate hydratase (TERG_06569) | 2.297647835 |
|  |  | Homocitrate synthase (TERG_07127) | 1.591953832 |
|  |  | Oligopeptide transporter (TERG_07783) | 1.59740161 |
|  |  | Isocitrate dehydrogenase (TERG_07814) | 2.490766563 |
|  |  | Amino acid transporter transmembrane domain-containing protein (TERG_07923) | 1.892882175 |
|  |  | Branched-chain amino acid aminotransferase (TERG_08300) | -2.150095917 |
| F | Nucleotide transport and metabolism | H+/nucleoside cotransporter (TERG_01084) | 2.727948527 |
|  |  | GMP synthase (TERG_01446) | 1.954306529 |
|  |  | Phosphoribosylaminoimidazolecarboxamide formyltransferase/IMP cyclohydrolase (TERG_07503) | 2.722331387 |
| G | Carbohydrate transport and metabolism | Uncharacterized protein (TERG_00608) | -1.717884938 |
|  |  | N-acetylglucosamine-6-phosphate deacetylase (TERG_03223) | 3.052239347 |
|  |  | Glucosamine-6-phosphate isomerase (TERG_03226) | 2.457980059 |
|  |  | Phosphotransferase (TERG_03269) | 2.82287982 |
|  |  | GPI anchored cell wall protein (TERG_04587) | -2.253259481 |
|  |  | Phosphoribosylglycinamide formyltransferase (TERG_06128) | 4.987810911 |
|  |  | Zn(2)-C6 fungal-type domain-containing protein (TERG_06884) | -2.064983875 |
|  |  | LysM domain-containing protein (TERG_08252) | -2.32108965 |
|  |  | Dihydroxyacetone kinase (TERG_08455) | -1.665690097 |
| H | Coenzyme transport and metabolism | Pyruvate decarboxylase (TERG_00355) | 2.303563312 |
|  |  | GTP cyclohydrolase 1 (GTP cyclohydrolase I) (TERG_04519) | 1.796545814 |
| I | Lipid transport and metabolism | Cytochrome P450 (TERG_00099) | -2.474337925 |
|  |  | AB hydrolase-1 domain-containing protein (TERG_00458) | 2.010759688 |
|  |  | Cytochrome P450 alkane hydroxylase (TERG_02747) | -2.148367307 |
|  |  | Sterol 24-C-methyltransferase (TERG_03102) | -2.882608015 |
|  |  | Bifunctional lycopene cyclase/phytoene synthase (TERG_03917) | 1.97962686 |
|  |  | Phenylacetyl-CoA ligase (TERG_04003) | -2.115549894 |
|  |  | Enoyl-CoA hydratase/isomerase (TERG_04250) | 2.266278539 |
|  |  | 3-methylcrotonyl-CoA carboxylase subunit alpha (TERG_04291) | 2.499363391 |
|  |  | Methylcrotonoyl-CoA carboxylase (TERG_04294) | 1.907038444 |
|  |  | SAM-dependent methyltransferase Erg6/SMT-type domain-containing protein (TERG_05431) | 3.081897891 |
|  |  | Peroxisomal hydratase-dehydrogenase-epimerase (TERG_05621) | -2.02447389 |
|  |  | SGNH hydrolase-type esterase domain-containing protein (TERG_06359) | -2.464004226 |
|  |  | Phosphatidic acid phosphatase type 2/haloperoxidase domain-containing protein (TERG_06778) | -2.204168343 |
|  |  | Endopolyphosphatase (TERG_07964) | -1.727586863 |
|  |  | Amidase domain-containing protein (TERG_07997) | -1.959121423 |
|  |  | Squalene/phytoene synthase (TERG_08447) | 2.7841668 |
|  |  | Fatty acid synthase subunit beta (TERG_08528) | 1.750271911 |
| J | Translation, ribosomal structure and biogenesis | Lysine--tRNA ligase (TERG_00063) | 1.769656013 |
|  |  | Glutamate--tRNA ligase (TERG_00363) | 3.209116676 |
|  |  | 2'-phosphotransferase (TERG_00427) | -2.020682885 |
|  |  | 60S ribosomal protein L8 (TERG_01102) | 1.715501677 |
|  |  | Ribosomal protein L24 (TERG_01415) | 1.759097747 |
|  |  | CCAAT-binding factor domain-containing protein (TERG_02021) | 1.527351292 |
|  |  | tRNA-binding domain-containing protein (TERG_02314) | 3.800122546 |
|  |  | Methyltransferase (TERG_03318) | 2.326413705 |
|  |  | Ribosomal RNA-processing protein 42 (TERG_03530) | 3.776116771 |
|  |  | Large ribosomal subunit protein uL5m (TERG_04057) | 2.001653553 |
|  |  | Large ribosomal subunit protein uL29m (TERG_04835) | 1.594860258 |
|  |  | tRNA-splicing endonuclease subunit Sen34 (TERG_05061) | 2.309188474 |
|  |  | Small ribosomal subunit protein uS5m (TERG_05110) | 2.083577309 |
|  |  | PEK protein kinase (TERG_05313) | -1.588870453 |
|  |  | RNB domain-containing protein (TERG_05739) | 2.506524089 |
|  |  | Uncharacterized protein (TERG_06618) | -1.79734489 |
|  |  | Elongation factor G (TERG_06917) | 2.13504757 |
|  |  | RNase III domain-containing protein (TERG_07806) | 2.119428284 |
|  |  | RNB domain-containing protein (TERG_07953) | 2.122824523 |
|  |  | Amidase domain-containing protein (TERG_07997) | -1.959121423 |
|  |  | 40S ribosomal protein S22 (TERG_08108) | 2.878511633 |
|  |  | Elongation factor EFG domain-containing protein (TERG_08491) | -1.764750464 |
| K | Transcription | MADS-box domain-containing protein (TERG_00139) | -1.585758548 |
|  |  | R3H domain-containing protein (TERG_00166) | -1.890317232 |
|  |  | Grh/CP2 DB domain-containing protein (TERG_00222) | 2.563334585 |
|  |  | BHLH domain-containing protein (TERG_00615) | -1.744052172 |
|  |  | Gfd2/YDR514C-like C-terminal domain-containing protein (TERG_00836) | 1.878209512 |
|  |  | Uncharacterized protein (TERG_00977) | 1.606614131 |
|  |  | DNA-directed RNA polymerase III subunit Rpc5 (TERG_02256) | 2.964594107 |
|  |  | Uncharacterized protein (TERG_02830) | -1.975652314 |
|  |  | Uncharacterized protein (TERG_02902) | -1.738143416 |
|  |  | Uncharacterized protein (TERG_05226) | -1.522786849 |
|  |  | Transcription factor domain-containing protein (TERG_05624) | -1.615571143 |
|  |  | MYB family conidiophore development protein FlbD (TERG_06164) | 2.331222358 |
|  |  | DNA-directed RNA polymerases I and III subunit RPAC1 (TERG_06305) | 4.353967738 |
|  |  | DUF7514 domain-containing protein (TERG_06875) | -1.699357749 |
|  |  | Deacetylase sirtuin-type domain-containing protein (TERG_07330) | -2.468492528 |
|  |  | tRNA ligase (TERG_07346) | -2.118484243 |
|  |  | HMG box domain-containing protein (TERG_08029) | 4.369829596 |
|  |  | BZIP domain-containing protein (TERG_08141) | -1.52473788 |
|  |  | BZIP domain-containing protein (TERG_08825) | -2.657624295 |
| L | Replication, recombination and repair | Uracil-DNA glycosylase (TERG_04652) | -1.834316857 |
|  |  | DNA mismatch repair protein MSH5 (TERG_07948) | -1.783479662 |
| M | Cell wall/membrane/envelope biogenesis | F-box domain-containing protein (TERG_08597) | -7.600317292 |
| O | Posttranslational modification, protein turnover, chaperones | Sulfhydryl oxidase (TERG_00164) | -1.659744061 |
|  |  | PCI domain-containing protein (TERG_00238) | -1.702220211 |
|  |  | Uncharacterized protein (TERG_00608) | -1.717884938 |
|  |  | Hsp70-like protein (TERG_01002) | 1.946697486 |
|  |  | glutathione transferase (TERG_01405) | -1.930958664 |
|  |  | Proteasome subunit beta (TERG_01596) | -1.642588481 |
|  |  | Subtilisin-like protease 4 (Sub4, TERG_01617) | 1.691234004 |
|  |  | Proteasome subunit beta (TERG_01662) | -1.516024435 |
|  |  | Proteasome component C5 (TERG_01664) | -2.491322792 |
|  |  | Proteasome subunit beta (TERG_02221) | -1.796728751 |
|  |  | Peptidase A1 domain-containing protein (TERG_02315) | 1.704935451 |
|  |  | RBR-type E3 ubiquitin transferase (TERG_02572) | -1.700415457 |
|  |  | C2H2 finger domain-containing protein (TERG_02634) | 1.918115678 |
|  |  | Proteasome endopeptidase complex (TERG_03371) | -1.749925022 |
|  |  | Octapeptidyl aminopeptidase (TERG_03374) | 2.352001338 |
|  |  | Subtilisin-like protease 3 (Sub3, TERG_03815) | 3.128729291 |
|  |  | J domain-containing protein (TERG_04087) | -1.653708934 |
|  |  | GPI anchored cell wall protein (TERG_04587) | -2.253259481 |
|  |  | Cell division control protein 48 (TERG_05378) | -1.761750904 |
|  |  | Leucine Aminopeptidase 1 (LAP1, TERG_05652) | 2.924446177 |
|  |  | Transglutaminase-like domain-containing protein (TERG_05842) | -2.703823674 |
|  |  | Peptide-methionine (S)-S-oxide reductase (TERG_06206) | 1.564651331 |
|  |  | Heat shock 70 kDa protein (TERG_06505) | -1.538721644 |
|  |  | Ubiquitin carboxyl-terminal hydrolase (TERG_06741) | -2.636039085 |
|  |  | Serine/threonine-protein kinase ATG1 (TERG_06765) | -1.613198748 |
|  |  | Hsp90-like protein (TERG_06963) | -1.799192167 |
|  |  | Metacaspase-1B (TERG_07319) | -1.903470346 |
| P | Inorganic ion transport and metabolism | H+/nucleoside cotransporter (TERG_01084) | 2.727948527 |
|  |  | CFEM domain-containing protein (TERG_01507) | 2.203949242 |
|  |  | Vacuolar calcium ion transporter (TERG_04766) | 1.964002642 |
|  |  | Manganese/iron superoxide dismutase C-terminal domain-containing protein (TERG_04819) | 3.489166305 |
|  |  | Leucine Aminopeptidase 1 (LAP1, TERG_05652) | 2.924446177 |
|  |  | Superoxide dismutase (TERG_07262) | 4.101449443 |
| Q | Secondary metabolites biosynthesis, transport and catabolism | Cytochrome P450 (TERG_00099) | -2.474337925 |
|  |  | Cytochrome P450 alkane hydroxylase (TERG_02747) | -2.148367307 |
|  |  | Cytochrome P450 oxidoreductase (TERG_03101) | -3.457078707 |
|  |  | ATP-dependent bile acid permease (TERG_04323) | -1.985616369 |
|  |  | Fe2OG dioxygenase domain-containing protein (TERG_06507) | -1.779494065 |
|  |  | Cytochrome P450 (TERG_06770) | -2.300440342 |
|  |  | Multicopper oxidase (TERG_07608) | -1.987136528 |
|  |  | ABC multidrug transporter (TERG_08299) | -1.862086422 |
|  |  | ABC multidrug transporter MDR2 (TERG_08613) | -2.255252487 |
| R | General function prediction only | Major facilitator superfamily profile domain-containing protein (TERG_00162) | -2.660754482 |
|  |  | Major facilitator superfamily profile domain-containing protein (TERG_00348) | 2.208637976 |
|  |  | THUMP domain-containing protein (TERG_00356) | 2.845215636 |
|  |  | RRM domain-containing protein (TERG_00938) | -1.666731452 |
|  |  | Polynucleotide 5'-hydroxyl-kinase GRC3 (TERG_00978) | 1.643620321 |
|  |  | G domain-containing protein (TERG_01167) | 1.927516653 |
|  |  | C2H2-type domain-containing protein (TERG_01313) | 1.564469089 |
|  |  | TIGR01456 family HAD hydrolase (TERG_01347) | 1.855177199 |
|  |  | CAAX prenyl protease (TERG_01583) | -1.588029884 |
|  |  | Fumarylacetoacetase-like C-terminal domain-containing protein (TERG_01661) | -2.355603548 |
|  |  | C2H2-type domain-containing protein (TERG_01956) | -2.245154242 |
|  |  | C2H2-type domain-containing protein (TERG_02061) | -1.937543988 |
|  |  | TIGR01456 family HAD hydrolase (TERG_02113) | 1.988902496 |
|  |  | Uncharacterized protein (TERG_02455) | -1.512168667 |
|  |  | WD repeat protein (TERG_02611) | 1.534318209 |
|  |  | Major facilitator superfamily profile domain-containing protein (TERG_02614) | 2.409608237 |
|  |  | Major facilitator superfamily profile domain-containing protein (TERG_02844) | -2.132641375 |
|  |  | Sterol 24-C-methyltransferase (TERG_03102) | -2.882608015 |
|  |  | Integral membrane protein (TERG_03122) | -1.785286692 |
|  |  | Siderochrome-iron transporter Sit1 (TERG_03174) | 2.000129077 |
|  |  | FAD-binding domain-containing protein (TERG_03218) | -1.635904572 |
|  |  | DUF221 domain-containing protein (TERG_03282) | 1.612092076 |
|  |  | C2H2-type domain-containing protein (TERG_03861) | -2.637896458 |
|  |  | VLRF1 domain-containing protein (TERG_03934) | -2.497194499 |
|  |  | DNA/RNA-binding domain-containing protein (TERG_04107) | -1.556069697 |
|  |  | Chromo domain-containing protein (TERG_04546) | 1.817517897 |
|  |  | Major facilitator superfamily profile domain-containing protein (TERG_04711) | 3.638277252 |
|  |  | C2H2-type domain-containing protein (TERG_04871) | 1.584612656 |
|  |  | Major facilitator superfamily profile domain-containing protein (TERG_05391) | 2.902063475 |
|  |  | SAM-dependent methyltransferase Erg6/SMT-type domain-containing protein (TERG_05431) | 3.081897891 |
|  |  | Leucine Aminopeptidase 1 (LAP1, TERG_05652) | 2.924446177 |
|  |  | AN1-type domain-containing protein (TERG_05655) | -1.692346958 |
|  |  | Atypical/ABC1/ABC1-C protein kinase (TERG_05702) | -2.192661396 |
|  |  | AmmeMemoRadiSam system protein B (TERG_05841) | -1.720888843 |
|  |  | Tr-type G domain-containing protein (TERG_05987) | -1.543137028 |
|  |  | WD repeat-containing protein (TERG_06121) | 4.474920019 |
|  |  | Fe2OG dioxygenase domain-containing protein (TERG_06507) | -1.779494065 |
|  |  | 3-ketoacyl-acyl carrier protein reductase (TERG_06998) | -1.82889315 |
|  |  | Enoyl reductase domain-containing protein (TERG_07017) | 2.702782021 |
|  |  | C2 domain-containing protein (TERG_07200) | 2.571172107 |
|  |  | C2H2-type domain-containing protein (TERG_07266) | 1.808829909 |
|  |  | Glycosyl transferase CAP10 domain-containing protein (TERG_07500) | 2.064568687 |
|  |  | Efflux pump dotC (Dothistromin biosynthesis protein C) (TERG_07539) | -2.541857572 |
|  |  | Major facilitator superfamily profile domain-containing protein (TERG_08095) | 1.857694137 |
| S | Function unknown | Sulfur metabolite repression control protein B (TERG_00539) | 1.755940728 |
|  |  | AMMECR1 domain-containing protein (TERG_00968) | -1.564874116 |
|  |  | TMEM205-like domain-containing protein (TERG_01037) | 2.283829611 |
|  |  | Proteasome component (TERG_01646) | -1.7997984 |
|  |  | Uncharacterized protein (TERG_02262) | -1.717471479 |
|  |  | Ubiquitinyl hydrolase 1 (TERG_02468) | -1.843965638 |
|  |  | Mitochondrial import inner membrane translocase subunit Tim21 (TERG_03151) | 2.733429246 |
|  |  | Bms1-type G domain-containing protein (TERG_03498) | 1.620041045 |
|  |  | Fermentation associated protein (TERG_04927) | -1.517579436 |
|  |  | Uncharacterized protein (TERG_04994) | 3.074424788 |
|  |  | Disease resistance R13L4/SHOC-2-like LRR domain-containing protein (TERG_05683) | -1.826483836 |
|  |  | Selenoprotein O (TERG_06473) | 2.734882218 |
|  |  | Uncharacterized protein (TERG_06713) | -2.156317806 |
|  |  | Proteasome activator subunit 4 (TERG_07227) | -2.146479428 |
|  |  | Nuclear envelope protein (TERG_07613) | 1.61494082 |
|  |  | PQ loop repeat protein (TERG_08593) | 2.02627261 |
| T | Signal transduction mechanisms | Guanine nucleotide-binding protein subunit beta-like protein (TERG_00783) | 1.695442885 |
|  |  | CFEM domain-containing protein (TERG_01507) | 2.203949242 |
|  |  | Class E vacuolar protein-sorting machinery protein HSE1 (TERG_03041) | -2.079910166 |
|  |  | Serine/threonine-protein kinase ATG1 (TERG_04042) | -1.83126305 |
|  |  | Bax Inhibitor family protein (TERG_04326) | -2.281832512 |
|  |  | Aminotransferase class I/classII large domain-containing protein (TERG_04543) | 1.763904134 |
|  |  | EGF-like domain-containing protein (TERG_05138) | 3.169137221 |
|  |  | Protein kinase domain-containing protein (TERG_05480) | -1.821576092 |
|  |  | Serine/threonine-protein kinase ATG1 (TERG_06765) | -1.613198748 |
|  |  | Protein phosphatase (TERG_06949) | 1.978281918 |
|  |  | Amidase domain-containing protein (TERG_07997) | -1.959121423 |
|  |  | Vacuolar protein sorting-associated protein 27 (TERG_08767) | -1.623307246 |
| U | Intracellular trafficking, secretion, and vesicular transport | Protein transport protein SEC22 (TERG_01244) | 2.686399697 |
|  |  | TLC domain-containing protein (TERG_05392) | 2.001968358 |
|  |  | Inositol polyphosphate-related phosphatase domain-containing protein (TERG_05707) | -1.539098669 |
|  |  | Serine/threonine-protein kinase ATG1 (TERG_06765) | -1.613198748 |
|  |  | Vacuolar protein 8 (TERG_08489) | -1.928503163 |
|  |  | Vacuolar protein sorting-associated protein 27 (TERG_08767) | -1.623307246 |
| V | Defense mechanisms | Allergen Asp f 7 (TERG_04582) | -1.949502108 |
|  |  | Protein kinase domain-containing protein (TERG_05480) | -1.821576092 |
|  |  | Endo-1,3(4)-beta-glucanase (TERG_06189) | -2.28627238 |
|  |  | Yeast cell wall synthesis Kre9/Knh1-like N-terminal domain-containing protein (TERG_06246) | -1.733596865 |
|  |  | Uncharacterized protein (TERG_06839) | 3.389981979 |
|  |  | Glycoprotein X (TERG_08225) | 1.720624203 |
| W | Extracellular structures | Endoglucanase (TERG_04504) | 1.958435674 |
|  |  | Allergen Asp f 7 (TERG_04582) | -1.949502108 |
|  |  | Endo-1,3(4)-beta-glucanase (TERG_06189) | -2.28627238 |
|  |  | Yeast cell wall synthesis Kre9/Knh1-like N-terminal domain-containing protein (TERG_06246) | -1.733596865 |
|  |  | Uncharacterized protein (TERG_06839) | 3.389981979 |
|  |  | Glycoprotein X (TERG_08225) | 1.720624203 |
| Y | Nuclear structure | Uncharacterized protein (TERG_00042) | 1.580529942 |
|  |  | Cell wall proline rich protein (TERG_01374) | -1.944609138 |
|  |  | Peroxin-14 (TERG_03510) | -1.72663323 |
|  |  | AAA+ ATPase domain-containing protein (TERG_03763) | -1.909835497 |
| Z | Cytoskeleton | Chromo domain-containing protein (TERG_04546) | 1.817517897 |
|  |  | AIG1-type G domain-containing protein (TERG_05617) | -3.907944275 |
|  |  | Actin (TERG_06637) | -1.629282027 |
|  |  | BZIP domain-containing protein (TERG_07733) | 1.742136526 |
|  |  | Translationally-controlled tumor protein homolog (TERG_08234) | 1.589660786 |
|  |  | Kinesin-like protein (TERG_08479) | -3.092370908 |

**Table S5: Validation of transcriptome using qRT-PCR.** Expression levels of selected genes were normalised to beta-actin as a housekeeping gene. PacC was used as a qRT-PCR internal control.

| **Gene** | **Transcriptomics (Log_2_FC)** | **qRT-PCR (Log_2_FC)** |
| --- | --- | --- |
| Subtilisin-like protease 4 (TERG_01617) (Sub4) | 1.691234004 | 3.7478 |
| Subtilisin-like protease 3 (TERG_03815) (Sub3) | 3.128729291 | 2.633394 |
| Leucine Aminopeptidase 1 (TERG_05652) (LAP1) | 2.924446177 | 0.998755 |
| Hsp90-like protein (TERG_06963) | -1.799192167 | -1.8740169 |
| Siderochrome-iron transporter Sit1 (TERG_03174) | 2.000129077 | 2.364354 |
| Sterol 24-C-methyltransferase (TERG_03102) | -2.882608015 | -2.90339 |
| SAM-dependent methyltransferase Erg6/SMT-type domain-containing protein (TERG_05431) | 3.081897891 | 5.063913995 |
| PacC (RT-Control) | - | 1.601978 |

**Supplementary References**

Bitencourt, T. A., Macedo, C., Franco, M. E., Assis, A. F., Komoto, T. T., Stehling, E. G., Beleboni, R. O., Malavazi, I., Marins, M., & Fachin, A. L. (2016). Transcription profile of Trichophyton rubrum conidia grown on keratin reveals the induction of an adhesin-like protein gene with a tandem repeat pattern. *BMC Genomics*, *17*, 249.

Deng, Z.-L., Münch, P. C., Mreches, R., & McHardy, A. C. (2022). Rapid and accurate identification of ribosomal RNA sequences via deep learning. Nucleic Acids Research, 50(10), e60.

Dobin, A., Davis, C. A., Schlesinger, F., Drenkow, J., Zaleski, C., Jha, S., Batut, P., Chaisson, M., & Gingeras, T. R. (2013). STAR: ultrafast universal RNA-seq aligner. Bioinformatics (Oxford, England), 29(1), 15–21.

Giddey, K., Favre, B., Quadroni, M., & Monod, M. (2007). Closely related dermatophyte species produce different patterns of secreted proteins. *FEMS Microbiology Letters*, *267*(1), 95–101.

Giddey, K., Monod, M., Barblan, J., Potts, A., Waridel, P., Zaugg, C., & Quadroni, M. (2007). Comprehensive analysis of proteins secreted by Trichophyton rubrum and Trichophyton violaceum under in vitro conditions. *Journal of Proteome Research*, *6*(8), 3081–3092.

Grigoriev, I. V., Nikitin, R., Haridas, S., Kuo, A., Ohm, R., Otillar, R., Riley, R., Salamov, A., Zhao, X., Korzeniewski, F., Smirnova, T., Nordberg, H., Dubchak, I., & Shabalov, I. (2014). MycoCosm portal: gearing up for 1000 fungal genomes. *Nucleic Acids Research*, *42*(Database issue), D699–D704.

Sriranganadane, D., Waridel, P., Salamin, K., Feuermann, M., Mignon, B., Staib, P., Neuhaus, J.-M., Quadroni, M., & Monod, M. (2011). Identification of novel secreted proteases during extracellular proteolysis by dermatophytes at acidic pH. *Proteomics*, *11*(22), 4422–4433.

Wick, R. R., Judd, L. M., & Holt, K. E. (2019). Performance of neural network basecalling tools for Oxford Nanopore sequencing. Genome Biology, 20(1), 129.
